# 25 Years of Electronic Health Record Implementation Processes: Scoping Review

**DOI:** 10.2196/60077

**Published:** 2025-03-03

**Authors:** Harriet Finnegan, Nicola Mountford

**Affiliations:** 1 School of Business Maynooth University Kildare Ireland

**Keywords:** electronic health record system, EHR, electronic medical record, EMR, scoping review, process, implementation

## Abstract

**Background:**

Electronic health record (EHR) systems have undergone substantial evolution over the past 25 years, transitioning from rudimentary digital repositories to sophisticated tools that are integral to modern health care delivery. These systems have the potential to increase efficiency and improve patient care. However, for these systems to reach their potential, we need to understand how the process of EHR implementation works.

**Objective:**

This scoping review aimed to examine the implementation process of EHRs from 1999 to 2024 and to articulate process-focused recommendations for future EHR implementations that build on this history of EHR research.

**Methods:**

We conducted a scoping literature review following a systematic methodological framework. A total of 5 databases were selected from the disciplines of medicine and business: EBSCO, PubMed, Embase, IEEE Explore, and Scopus. The search included studies published from 1999 to 2024 that addressed the process of implementing an EHR. Keywords included “EHR,” “EHRS,” “Electronic Health Record*,” “EMR,” “EMRS,” “Electronic Medical Record*,” “implemen*,” and “process.” The findings were reported in accordance with the PRISMA-ScR (Preferred Reporting Items for Systematic Reviews and Meta-Analyses Extension for Scoping Reviews) checklist. The selected literature was thematically coded using NVivo qualitative analysis software, with the results reported qualitatively.

**Results:**

This review included 90 studies that described the process of EHR implementation in different settings. The studies identified key elements, such as the role of the government and vendors, the importance of communication and relationships, the provision of training and support, and the implementation approach and cost. Four process-related categories emerged from these results: compliance processes, collaboration processes, competence-development processes, and process costs.

**Conclusions:**

Although EHRs hold immense promise in improving patient care, enhancing research capabilities, and optimizing health care efficiency, there is a pressing need to examine the actual implementation process to understand how to approach implementation. Our findings offer 7 process-focused recommendations for EHR implementation formed from analysis of the selected literature.

## Introduction

### Background

Over the past 25 years, electronic health record (EHR) systems have undergone remarkable development, becoming an important element of modern health care [1]. Since their inception in the late 20th century, EHRs have advanced substantially, propelled by both technological innovations and critical policy reforms [2]. Key legislation, such as the Health Information Technology for Economic and Clinical Health (HITECH) Act of 2009, was instrumental in accelerating the widespread adoption of EHRs, solidifying their role in enhancing health care delivery. Today, EHRs are indispensable for improving patient safety, improving operational efficiency, and ensuring that vital patient information is securely stored and easily accessible across health care settings [3].

Research to date has examined some of the key pre-implementation indicators of EHR adoption and success. Studies have revealed how certain organizational characteristics are likely to predict success, such as the size of the organization [4,5] and where it is located [6,7]. Other research has provided important details on postimplementation evaluations by users. These evaluations may include an increase or a decrease in the difficulty of tasks for the physician [8], the impact on patient care [9], and effects on privacy and security [10].

There is an extensive body of literature reviews published over the past 2 decades that explore the success factors and challenges associated with EHR implementation [11-20]. This has substantially and importantly improved our understanding of those factors that must be considered when planning EHR implementation. Fennelly et al [20], for example, identified 15 interlinked organizational, human, and technological factors that affect successful EHR implementation across primary, secondary, and long-term care settings. Our study built on this body of work by returning to the source literature with a process focus—digging beneath the success factors and challenges to examine the underpinning processes of EHR implementation. A process focus allows us to examine the connections between the factors already identified, unveiling new aspects, such as flow, activity, and temporality [21]. We, therefore, foregrounded and synthesized those papers that center on this vital process of *implementing* an EHR rather than delving into the technical intricacies of EHR technology itself.

### Defining an EHR

The World Health Organization defines the EHR as “a longitudinal record of patient health information generated by one or more encounters in any care delivery setting” [22]. These records may include details such as demographics, progress notes, vital signs, medications, immunizations, lab results, and radiology reports, which all provide a comprehensive view of a patient’s health. Similarly, the Centers for Medicare and Medicaid Services (CMS) emphasizes that EHRs play a crucial role in helping health care providers maintain accurate and up-to-date patient data over time, ensuring that key clinical and administrative information is easily accessible and securely shared among authorized users [23].

Electronic medical records (EMRs), in contrast, are records created by practitioners for specific encounters, examples of which may be hospital visits or the use of facilities within ambulatory environments. Finally, a personal health record (PHR) is data controlled by the patient through the use of an electronic application that they are able to provide to their health practitioners. PHRs support patient-centered health care by making medical records and other relevant information accessible to patients, assisting patients in health self-management [24]. We focused exclusively on EHRs rather than EMRs or PHRs. The scope of EHRs is generally larger than that of EMRs or PHRs. They require a broad range of data types and need to be able to connect these across systems, whereas EMRs are generally confined to an individual practice [25], limiting their scope, and PHRs are generally subject to personal management [26]. This makes them less complex, and therefore less interesting from a research perspective, than EHRs. EHRs require higher levels of interoperability, regulatory challenges, stakeholder involvement, and cost and time investment than EMRs or PHRs, making them the ideal focus of this research.

Although the terms “EHR” and “EMR” are conceptually distinct, they are often used interchangeably in the literature. We recognize that the definitions we used for EHRs and EMRs in our review are not universally observed, and the terminology used in the literature and in practice often reflects the contexts in which these systems are implemented rather than the strict definitional boundaries placed upon them. In instances where studies, such as Felt-Lisk et al [27], have examined systems referred to as EHRs but may have operationally aligned with our definition of EMRs, we opted for a more inclusive approach. This definitional ambiguity may mean that some of our recommendations may equally apply to systems labeled as EMRs rather than EHRs. Addressing this overlap is essential for advancing a more unified understanding of electronic records in health care; however, it was not the purpose of this research.

### Implementation Process

Rather than the broader issue of EHR adoption, which relates to the widespread acceptance and use of the technology across health care settings, our focus was on implementation as the practical, often complex, process of integrating EHR technology into health care environments. Adoption is the “phase of investigation, research, consideration and decision making in order to introduce a new innovation into the organization” [28,29]. Implementation is the “phase of internal strategy formation, project definition and activities in which an adopted application is introduced within the organization, with the aim of removing reservations and stimulating the optimum use of the application” [29]. Although adoption can occur both prior to and after implementation, these terms describe separate, distinct actions. We discussed EHRs in terms of the process of implementation, as defined by Bouwman et al [29].

### Study Focus

This scoping review examined the EHR implementation process over the past 25 years. The paper presented a qualitative thematic analysis of 90 relevant academic papers describing the EHR implementation process. Our review of 25 years’ worth of EHR implementation processes ultimately offers some advice and hope for more effective EHR implementations and those policies that support them.

## Methods

### Overview

A scoping review was conducted according to the 5-stage framework by Arksey and O’Malley [30]. Results were reported according to the PRISMA-ScR (Preferred Reporting Items for Systematic Reviews and Meta-Analyses Extension for Scoping Reviews) checklist (Multimedia Appendix 1).

#### Stage 1: Identifying the Research Question

Our research question was as follows: What have we learned about the process of implementing EHRs over the past 25 years?

#### Stage 2: Identifying Relevant Studies

A systematic literature search was conducted across 5 databases to identify all relevant literature: EBSCO, PubMed, EMBASE, IEEE Explore, and Scopus. The following specific keywords were used in the search strategy: (EHR OR EHRS OR Electronic Health Record* OR EMR OR EMRS OR Electronic Medical Record*) AND implemen* AND process (all in abstract). Detailed search strategies are provided in Multimedia Appendix 2. The research period was from January 1999 to August 2024 in line with the focus of this special issue looking at the past 25 years. We chose to include the word “process” in our search string, in addition to variations of the word “implement” in order to refine our results to include discussions focused on the process of implementing EHRs rather than those centered on the outcomes of EHR implementation. We also chose to search for the term “electronic medical records,” in addition to EHRs in the initial stage, even though the focus of our paper was on EHRs. This inclusion of EMRs in the initial search allowed us to account for discrepancies in the language used when discussing EHRs. This initial inclusion in our search criteria also meant we were able to manually exclude EMR studies that were purely discussions of EMRs and manually include EMR studies that also discussed EHRs or possessed the same functionalities of an EHR. This broadening of our search terms meant that our analysis of the literature was more thorough. We chose only empirical studies because we sought evidence of specific experiences of EHR implementation processes.

#### Inclusion Criteria

Studies meeting the following inclusion criteria were included: (1) published from January 1999 to August 2024, (2) peer reviewed, (3) journal papers, (4) published in English, and (5) mentioned the implementation process of EHRs.

#### Exclusion Criteria

Studies meeting the following criteria were excluded: (1) focused purely on the aftermath of implementation; (2) did not discuss the implementation process; (3) did not clearly report methods; (4) nonempirical; (5) not published in a peer-reviewed journal; (6) letters to the editor, editorials, or essays; (7) gray literature and review papers; and (8) discussed single-location EMRs.

#### Stage 3: Study Selection

All citations were uploaded to Covidence, a web-based research tool used by researchers to collaborate and organize citations in systematic reviews. Duplicates were removed automatically by the software and manually by both authors. Both authors screened all 4454 remaining papers by title and abstract and 226 papers by full text. Both authors also reviewed any disagreements before proceeding to the next stage of screening. This was done at each stage of screening to ensure consistency between decisions. Any disagreements were resolved by both authors discussing the eligibility of the paper in relation to the research focus and the agreed-upon inclusion and exclusion criteria until reaching consensus.

#### Stage 4: Charting the Data

Data were extracted by the first author using Microsoft Excel, including the following details: author(s)/publication year, country of origin, aim(s)/purpose, study design, type of organization, study population/sample size, record type, and methods.

#### Stage 5: Collating, Summarizing, and Reporting Results

After reviewing all full texts of the eligible studies, the first author loaded the remaining 90 full texts into NVivo, a qualitative data analysis software program. This software was used to manually organize the texts and facilitate the thematic coding of the data. All 90 full texts were then thematically coded by the first author. The second author thematically coded a subset of 10 (11.1%) full texts. Both authors then met to discuss any differences in coding decisions. This was carried out to ensure intercoder reliability.

## Results

### Sample Characteristics

**Figure 1:**
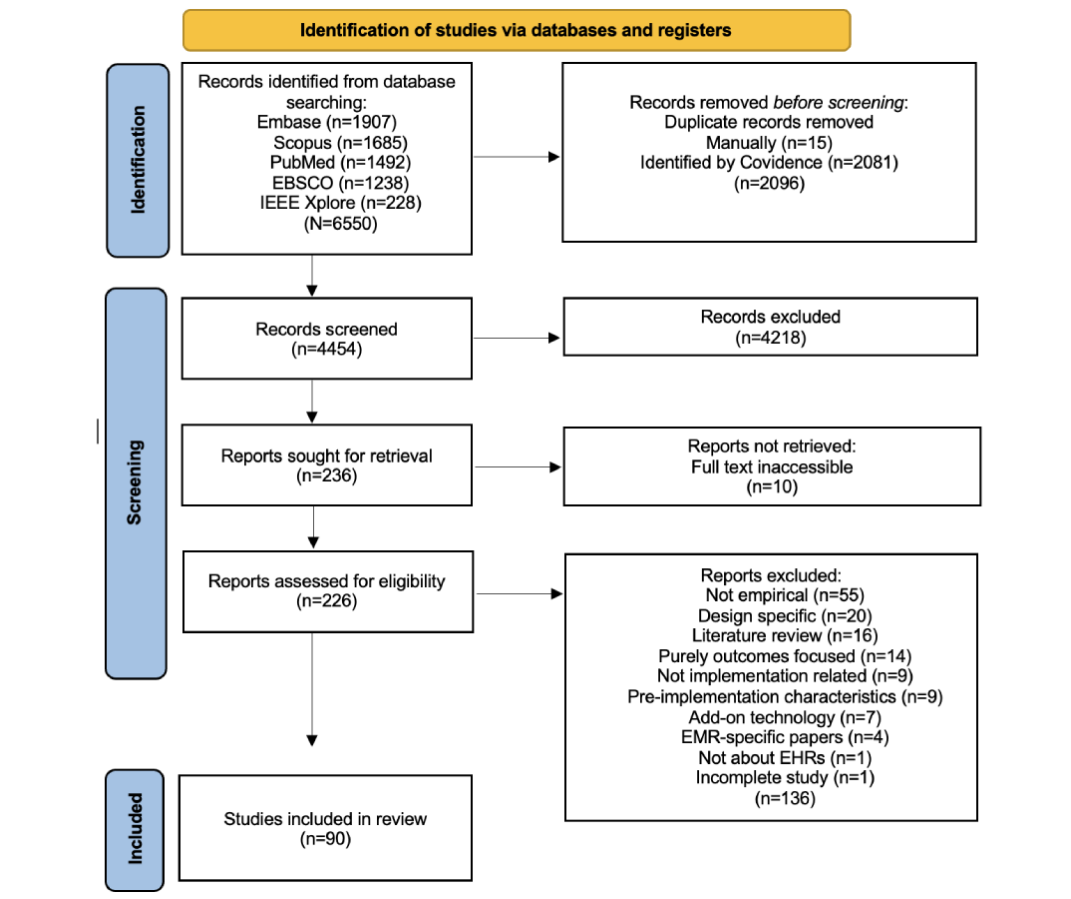
PRISMA-ScR flow diagram showing the study selection process. PRISMA-ScR: Preferred Reporting Items for Systematic Reviews and Meta-Analyses Extension for Scoping Reviews.

**Figure 2:**
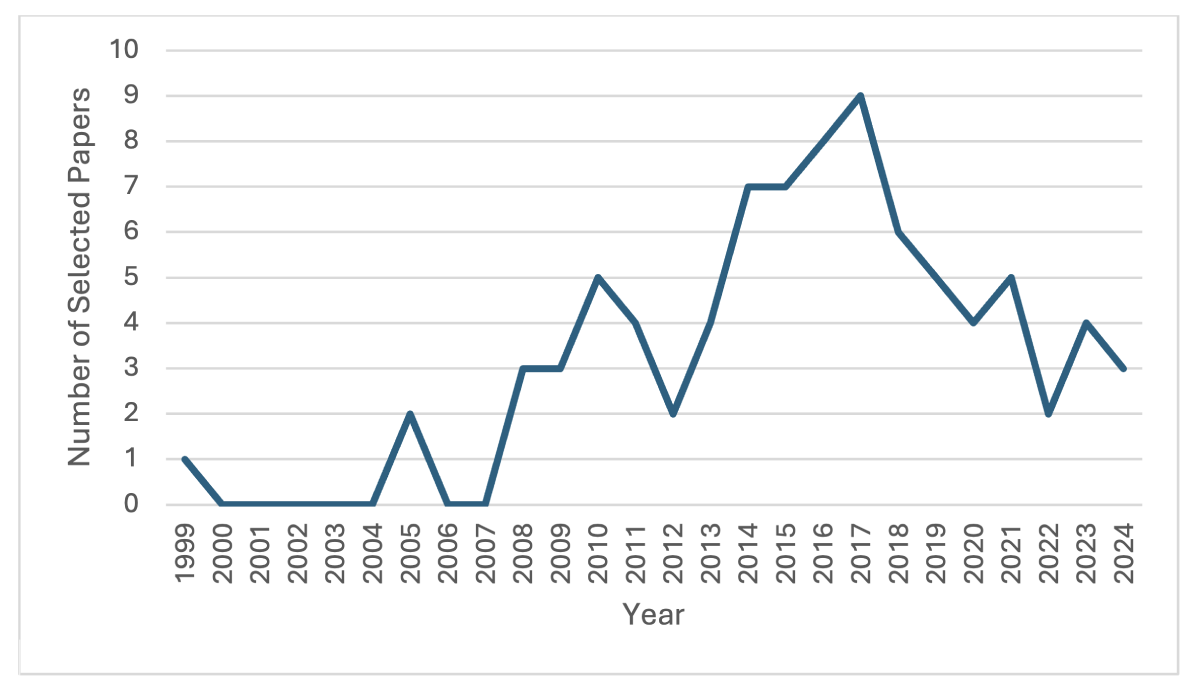
Distribution of the selected papers by year of publication.

The paper-screening process is illustrated in detail in Figure 1, while the distribution of the selected studies by year of publication is shown in Figure 2. Among the 90 papers, some discussed more than 1 country in their study. This included 37 (41.1%) studies conducted in the United States, 11 (12.2%) in England, 6 (6.7%) in Australia, 5 (5.6%) in Germany, 5 (5.6%) in Denmark, 3 (3.3%) in the Netherlands, 3 (3.3%) in Italy, 3 (3.3%) in Canada, 3 (3.3%) in Norway, 2 (2.2%) in Singapore, 2 (2.2%) in Kenya, 2 (2.2%) in the United Kingdom, and the remaining countries mentioned in only 1 (1.1%) study each. The majority of studies (n=59, 65.6%) were qualitative, while the remainder (n=19, 21.1%) were quantitative or used mixed methods (n=12, 13.3%). The selected papers begin in 1999 with 1 publication. Examining these figures showed initial inactivity on the topic, followed by steady growth starting in 2008 and peaking in 2017, with a slight decline in recent years. The peak years from 2014 to 2017 suggest a strong research interest during this time. Recently, from 2019 to 2024, there seemed to be a stabilization in research output, with around 4 publications annually. The main characteristics of the included studies are presented in Multimedia Appendix 3.

During the thematic coding process, sections of text within each paper were allocated to specific codes rather than entire papers being allocated to one code. As a result, multiple different codes may have appeared in one paper, as can be seen in the coding distribution table (Table 1). The full coding structure is provided in Multimedia Appendix 4.

**Table 1:** Coding distribution across studies (N=90).

Theme and codes			Studies, n (%)		References
**Compliance**					
	Government	29 (32.2)		[27,31-58]	
	Policy	21 (23.3)		[31,35,37-41,44,46,48,49,52,55,58-65]	
	Regulations	10 (11.2)		[31,34,36,55,62,63,65-68]	
	Vendors	20 (22.2)		[27,37,38,49,53,59,60,67,69-80]	
**Collaboration**					
	Communication	40 (44.4)		[32-34,36-38,40-42,44-46,49-51,59-61,63,66,68,70,71,74-76,78,79,81-92]	
	Relationships	30 (33.3)		[32-36,40,44,48-50,57,60,65,66,68-71,76,81,84,85,89,91-97]	
**Competence**					
	Training	44 (48.9)		[31,33,38-42,46,48,50,53,54,59-61,63,64,66-70,76,77,79,81-83,86,89,90,94-106]	
	Support	35 (38.9)		[27,32,36,39,40,42,43,45,48,50,53,54,60,66,67,69-71,74-76,79,81,82,84,86,90,95,97,105-110]	
**Cost**					
	Cost	41 (45.6)		[27,31,33,35-40,42,43,46,48-50,52,54,56,60,63,65,66,68,69,71,72,75,82,88,90,92-94,96,97,106,108,110-113]	

### The Implementation Process

#### Overview

The 90 studies provide a broad overview of the process of EHR implementation over the past 25 years.

A process-based view considers time as a key element. EHR implementation is described by Boonstra et al [34] as a complex and time consuming process, and by Hernández-Ávila et al [48] as a gradual and often slow process. The main benefits of EHRs are likely to accrue in the long term, so it is important to envision them as long-term change management endeavors [96]. These long-term benefits may never be realized, however, where short-term time pressures jeopardize implementation. This may arise where there is limited time available to adapt the system to local conditions [98] or where the pace of implementation is being dictated by other ongoing projects [49]. Deadlines imposed by external actors may also result in implementation timelines being rushed. For example, political considerations may frame procurement arrangements [52], or vendors might enforce tight deadlines [73]. Diffusion processes require good planning and consume both time and resources [110]. Time looms large in our reviewed studies, justifying our process focus. There is a constant pressure of time in relation to the implementation [38], but the reality is that pre-existing day-to-day pressures may limit the pace of implementation [27].

Four central process-related categories emerged from the thematic review: (1) compliance processes featured indirectly through references to the important role of the government, policy, regulations, and vendors; (2) collaboration processes centered on the work of managing these implementations through communication and relationships; (3) competence-building processes included discussions of training and support; and (4) process costs drew attention to areas of cost.

#### Compliance Processes in EHR Implementation

Our review suggested that compliance processes in EHR implementation center around the government, policy, regulations, and vendors. The literature referenced government-related processes in 29 (32.2%) papers. There were discussions of policy-related processes in 21 (23.3%) papers. Regulation-related processes were considered in 10 (11.1%) papers, and vendor-related processes were referenced throughout 20 (22.2%) papers.

### Government-Related Compliance Processes

EHR implementation problems often arise at the national level rather than at regional or health service–specific levels. This is a result of the increased complexity of national implementation [38]. Nationwide implementation requires a huge change from stakeholders [42]. In a national project, practitioners operating in public and private domains require different engagement strategies to secure their buy-in [51]. A national strategy is, therefore, needed for a national EHR implementation process [110]. National EHRs require adaptation with clinician practices nationwide to ensure workflow processes are consistent [91]. Further recommendations from the literature include changing from a top-down implementation model to increased involvement of local organizations in decision-making [35]. Planners need to ensure potentially unclear areas are clarified with program management and that clinicians are informed and consulted [51]. National-level implementation is not just simple system installation; it is discovering ideas from private institutions and using them to drive best practices across the system nationwide [49]. Bottom-up implementation is, however, time-consuming and may hinder future collaborations [94], whereas the top-down nature of some projects contributes to a lack of organizational and user involvement in decision-making [52].

The role of the government in initiating and maintaining momentum around EHR implementation processes came through strongly from our review. Government leadership is considered a strategic advantage when the goal is the sustained enforcement of EHR initiatives [31]. Mature EHR systems benefit from being well integrated into the national health-planning documents of the government [39], while national reimbursement policies can increase EHR dissemination [49]. To maintain momentum, government commitment must be both strong and continued and accompanied by political support [36]. eHealth experts are, therefore, generally in favor of a strong central solution for political regulation problems [62]. It is, however, not just the national government that matters. Support from the local government also plays a crucial role in terms of finances, provision of resources, and technical support [41]. Indeed, implementation processes suffer where they lack sufficient coordination with the local government [41]. However, strong national or local government policies mandating the use of specific eHealth solutions may support EHR implementation processes [41]. Excessive regulation may also hinder the long-term sustainability of EHR initiatives [31].

Changes in the government may lead to having to modify an overall implementation process [35]. Shifts in the government strategy affect the power dynamics between national branches of large IT companies compared to national information and communication technology companies [55]. A change in the government also has the potential to result in uncertainty about the future of national programs [52,58]. It is, therefore, advisable for system evaluators to form close relationships with policy makers [35]. Alternatively, if there is a lack of a government-level information policy at the time of design and development, the sustainability of the EHR implementation is endangered [48].

### The Role of Vendors

Governments are not, however, the only organizational actors who can manage or lead EHR compliance processes. Vendor organizations that have strong centralized administrative and medical structures are drivers of organizational policies and processes that are crucial to compliance [64]. Indeed, in some instances, large private vendors may enter a market specifically to provide unified access to health care data [49]. It is the process of compliance of large technology companies with uniform national standards and rules that enables EHR implementation in this instance [55,60].

Vendor recommendations influence software choice processes [69]. EHR implementors cannot simply buy from the same vendor as their existing financial system and assume turnkey, seamless interoperability [71]. There are often limitations to existing vendor-based EHRs when compared with in-house systems, including autonomy of practice decisions at each hospital [70]. These limitations are also seen in difficult-to-assess EHR usability as a result of restrictive vendor license agreements [59].

EHR vendors are generally at least partly responsible for the amount of training received by users [69] and sometimes also responsible for providing technical support [27]. Indeed, vendors may use the provision of ongoing technical support as a form of training [77]. Both peer and technical support may be issued from these vendors, which can help end-users optimize their use of the EHRs and solve issues [67]. Even large vendors may need to consider EHR adaptations for small practices as part of the sale of the EHR, ensuring that a person within the practice conducts training and adapts it to the specific needs of the practice rather than relying on training by IT specialists [53].

#### Collaboration Processes in EHR Implementation

Collaboration in the literature includes communication and relationships throughout the management of the implementation process. Communication was mentioned in 40 (44.4%) papers. Relationships were discussed in 30 (33.3%) papers.

Collaboration among team members is important [60], as is communicating clear expectations and guidelines [61]. Consistent, reliable communication fosters trust [83], and well-articulated visions are important for the management of expectations [74]. Strong communication practices are an enabler of successful implementation outcomes [84]. However, a lack of communication during planning can cause issues [49].

Collaborating facilitates successful EHR implementation [95]. Cuccinello et al [36] illustrate this in their study of a vendor’s collaborative relationship with a health care department in Italy. Relationships with vendors build confidence within the organization and ensure strong external support [60]. Where, however, communication breaks down, it can jeopardize implementation processes [41]. Relationships between contractors and suppliers may become more impersonal and distant as a result [34]. Direct and close channels of communication between the implementer hospitals and software suppliers are, therefore, essential from the outset [89]. Kiepek and Sengstack [84] suggest an open and transparent relationship with external support from vendors, beginning with initial negotiations.

#### Competence-Building Processes in EHR Implementation

Competence building in the literature includes training and technical support. The literature referenced training in 44 (48.9%) papers. Support was discussed in 35 (38.9%) of the selected papers.

Training is necessary for successful EHR implementation [95]. This training should provide practitioners with the skills necessary to operate the system, as well as the confidence to help them adapt to the new system [98]. Sufficient training for practitioners is associated with improved well-being [100] and has a positive and substantial influence on perceived ease of use [67]. Hiring experts can help, for example, in providing technical support both during the implementation and afterward [110], and hiring clinical informaticists can help support EHR implementation and sustainment [86].

Issues surrounding training and implementation processes are numerous. These include a lack of resources for EHR training [70], the need to train medical personnel [38], a lack of training for smaller entities [38], ensuring sufficient time for staff training [41], providers and patients not receiving adequate support and training [63], and a lack of appropriately specific training [102]. The need for sufficient trained personnel is felt across all stages of an implementation process [39]. Staff that are not trained to interact with an EHR can hinder implementation [31,42]. Recommendations to enhance EHR training include ensuring that it occurs pre- and postimplementation on a continued cycle [86]. Decreased support is an additional issue [70], whether that be in relation to practitioner support [43] or in determining the right type of IT support for successful implementation [97].

#### Process Costs in EHR Implementation

Cost considerations were shown to be an important part of the implementation process, with 41 (45.6%) papers making reference to cost. Adoption of appropriate processes is crucial regarding system development time and budget [42]. As such, successful implementation should have sustainable funding that aligns with a national strategy for eHealth [41]. Determining costs and measures of success is a vital part of project management, especially in pre-implementation [108]. The role of monetary incentives in this stage of the process is also an enabler [36]. One of the challenges of a national project is that funding sources depend largely on the government [31]. Implementing a system on a national scale is an extremely complex activity [52]. It is difficult to manage, costly to maintain, and hard to sustain [89]. Financially, the most serious obstacle in implementing EHRs is the cost of electrification [38]. Further customization also leads to increased maintenance costs [60]. The high upfront cost of EHRs for small practices is a major factor limiting their use [27]. Most hospitals report substantial financial challenges in EHR implementation and use, including EHR and broadband implementation costs and the limited availability of grants and loans to support EHR implementation and use [113]. A lack of capital resources can hinder the EHR implementation process [43]. Other issues include inadequate capital for investment and maintenance costs [110], large-scale procurement being undertaken to save costs [52], attempts to implement EHRs halting due to financial issues [54], and the high costs of implementing a system [97].

## Discussion

### Principal Findings

This scoping review revealed 4 main areas for consideration in the EHR implementation process. These areas are compliance, collaboration, competence, and costs. Specific issues recurred in each area throughout the literature: the role of the government and the role of vendors in compliance processes, the importance of communication and relationships to facilitate collaboration processes, training and support to build competence; and the cost of financing throughout the implementation process. Many of the 19 interventions identified by Boonstra et al [13], and the 15 factors identified by Fennelly et al [20] pertaining to successful or effective implementation strategies featured within our findings. Rather than replicate the reviews of Fennelly et al [20] or Boonstra et al [13], however, we built on these to provide a focused exploration of the implementation process to complement the broader insights that these reviews offer. We built on these works to offer practical recommendations for use in the implementation process.

Our study and research question focused on the *process* of implementation of EHRs. The process of implementation deserves attention, especially when we consider that the implementation process influences implementation outcomes. Put differently, it is not just what system we implement, nor the recognition of challenges or facilitators, but also the process by which we navigate and manage these over time that ultimately decides how successful an EHR might be. There is, therefore, a need to understand how to approach these implementation processes in a way that is informed by previous implementations and the appropriate literature. To address this, we provide recommendations produced from our synthesis of the selected literature reviewed in this paper. Organizing our findings into compliance processes, collaboration processes, competence processes, and process costs allowed us to provide distinct actionable recommendations for stakeholders. Our recommendations emphasize implementation priorities in a way that facilitates targeted interventions. Our recommendations not only reaffirm established principles found in reviews, such as those by Boonstra et al [13] and Fennelly et al [20], but also provide a modernized roadmap for undertaking the implementation process.

#### Seven Process-Based Recommendations for EHR Implementation

The literature consistently demonstrates the value of both the government and vendors in ensuring sustained EHR compliance processes, while highlighting some dangers. We drew from this some recommendations for successful compliance processes. The first recommendation is to *maintain close and ongoing government/implementor relationships to balance user, government, and organizational requirements in the short and longer terms.* There is a need for the central government to work with hospitals and local governments to ensure EHRs satisfy the requirements of users [49]. Government planners have the power to exert more influence on public health care providers than private providers [51]. Liaising with organizations and policy makers to inform strategic decisions and policy making is important [35]. As part of this, it is necessary for evaluators of EHRs to form close relationships with policy makers [35]. The second recommendation is to *rebalance vendor/implementor relationships to ensure small-site customization and training that will drive sustained compliance.* The role of the vendor is notable, with numerous best-practice sites viewing their vendor as an active partner in the implementation and compliance process [76]. Designating key contact people to act as liaisons may help foster this relationship from the beginning. Fostering open, regular communication between vendors and implementers can also be done through regular meetings.

The literature clearly highlights the importance of collaboration processes across teams, as well as multistakeholder communication, in ensuring sustained support for EHR implementation. Breakdown of communication and relationships is damaging to implementation processes. We built on these findings to suggest some collaboration processes that support EHR implementation*.* Our third recommendation, therefore, is to *cultivate varied sources of support across stakeholder groups*. Strong and continued commitment and support at the highest level facilitate and support collaboration processes [36]. The types of support needed vary to include political support [36], practitioner support [108], and social support [109]. Supporting the interest in EHRs is an important behavior linked to successful EHR implementation [95]. Indeed, issues may arise where there is inadequate patient and broader community engagement around EHR implementation [88]. The fourth recommendation is to *pay particular attention to communication and collaboration in the implementation-planning phase of EHR implementation, including the development of cross-functional teams, the appointment of “opinion leaders’” and realistic envisioning of postimplementation challenges and benefits*. Effective communication processes are critical in an organization when implementing an EHR [70], especially during the implementation-planning phase [76]. Stakeholders working toward change also need a close collaborative working environment [65]. Forming cross-functional teams [60] and enlisting the collaborative effort of physicians, hospital administrators, IT specialists, and state ofﬁcials are integral to the process of design and development [48]. Appointing “opinion leaders” to hear concerns of practitioners makes audiences more receptive to implementation [45]. Crafting communication campaigns that balance potential expected benefits with realistic expectations of the challenges faced may be an issue [59]. Anticipating the challenges that will be faced during implementation can cause these issues, as each implementation occurs in a unique environment.

The studies clearly show the importance of competence-building processes, such as training and technical support provision during the implementation process. We drew from this to suggest competence-building processes that will positively contribute to the EHR implementation process. The fifth recommendation is to *ensure that staff are adequately trained to use the systems and for planners to consider the timing of this training when organizing wider implementation processes.* Implementation processes depend on training the staff who will be using the system [77]. Ongoing training and optimization are necessary [103], as well as curricula for EHR training [104]. Training processes must be ongoing, embedded in workflows, and flexible so that they can be tailored to the diverse needs of users [89]. In terms of timing, training should be conducted close to the time of actual implementation of new technology [54], with the most successful training session conducted within a few weeks of the system going live [76]. Some authors recommend that staff be required to complete training by the end of the preparatory phase to retain their access to the EHR [94]. Others suggest sending staff to training classes customized by job role, with trainers on-site for 1-2 weeks after the system goes live [64]. The time it takes practitioners to chart should be addressed early on in training [53], and training focusing on how the EHR will work should be replaced with a focus on how the EHR can be adapted to the practitioners [53]. Vadillo et al [54] stress the importance of providing proper training in basic computer functions, with training conducted in the classroom with an instructor rather than one to one. Recommendations also suggest that senior management provide practice leaders with IT training and have them visit an EHR-based practice [97]. The sixth recommendation is to *assess what support will be needed at each stage of the implementation and ensure that this support is put in place for an appropriate amount of time*. Support is critical during the go-live period [76]. Carayon et al [69] suggest having support staff present from the EHR vendor on the day the EHR goes live and having an expert user present at the clinic for the following weeks as a useful support to implementation processes. The availability of “super users” who offer support at go-live time is also noted as appropriate [60] and particularly useful when considering user support as a higher priority than initial user training [39]. Other notable forms of support are vendor support [60] and industry support [42]. Informal support (provided via Facebook, involving both vendors and peer-to-peer support) is also noted to be effective and efficient [74]. Support throughout the planning and implementation period ensures clarity of roles, strong communication practices, and a successful outcome [84].

Finally, the literature clearly demonstrates the integral role of cost throughout implementation. We drew from this discussion of cost in the literature to provide our concluding recommendation. The seventh recommendation is to *promote an understanding of the system as a long-term investment*. Some of the literature promotes the development process of a solid government reimbursement plan [49]. However, more central to our recommendation is deRiel et al’s study [39]. The authors discuss the importance of understanding the system, its value, and the total cost of ownership so that investments are not seen as one-time expenses but ongoing investments. Other authors suggest that the process of choosing an EHR system should center on its potential for improving clinical care rather than achieving cost savings [96]. Inadequate practitioner consultation processes [46], delays [58], and tendering processes all increase the cost of the overall process [34].

Our findings illustrate that successful implementation processes benefit from meticulous planning [76]. Our recommended processes for addressing compliance, collaboration, competence, and costs within the wider EHR implementation process aim to provide the materials for informed “meticulous *process* planning” to occur. Examining EHR implementation purely from a pre- versus postimplementation perspective of outcomes may blind both researchers and practitioners to the importance of the intervening implementation processes. Our review connects previously established barriers and facilitators with a time-and-action focus to offer additional insights into the process of implementation.

### Limitations

Several limitations should be considered in this scoping review. This study focused on EHRs and did not consider the implementation processes of other eHealth systems, which may benefit from future analysis. Our findings also highlight the need for improved standardization in the terminology surrounding electronic records in health care to better differentiate between EHRs and EMRs in both research and practice. This improved clarity would allow for greater comparability across studies and guide more tailored implementation processes. The included studies tended toward the United States and Europe, centering our analysis in the Global North. The search strategy included studies from 1999 onward. The resulting studies selected were mostly from 2007 onward. It is unlikely that this date limitation largely impacted the selected studies; however. it is possible that due to the date limitation, this scoping review may have missed interesting studies conducted prior to 1999. Finally, our specific search terms, chosen with the intention of focusing the literature on the implementation process, may mean that the literature describing the same event but not using those specific search terms was not retrieved. Additionally, shifts in the language and naming of systems in health care mean that this study may apply to systems discussed under a name other than “EHRs,” which have not been retrieved during the search strategy of this research. As such, we did not retrieve or include any studies discussing EHRs under the recently emerging term of “digital health records.”

### Conclusion

Completed implementation of EHRs is integral to improving health care delivery. The findings from this scoping review offer important insights into the complexities of the actual process of implementation and its subprocesses. Our review identified 3 key processes (compliance, collaboration, and competence building), as well as considering overall process costs. In doing so, we offered a new time- and action-based perspective on EHR implementation. Compliance processes reference the role of the government, policy, regulation, and vendors in shaping the implementation process. Collaboration processes promote the need for strong communication and the building of relationships across all stakeholders involved in the implementation process. Competence-building processes focus on ensuring that users are provided with the resources to be able to operate an EHR, centering around the importance of the timing of training and support. Finally, our discussion of process cost illustrates the importance of a time-focused financial approach during the implementation process.

We proposed 7 strategies in this review, which all provide a structured approach to navigating the different areas of implementation. Future research should focus on deepening our understanding of how these outlined strategies change and operate at different stages of the implementation process. This scoping review contributes to the intersection of management and health care research. We hope that the review results and recommended strategies provided will inform areas for future research and help develop future implementation processes.

## Appendix

Multimedia Appendix 1 PRISMA-ScR (Preferred Reporting Items for Systematic Reviews and Meta-Analyses Extension for Scoping Reviews) checklist.

Multimedia Appendix 2 Search terms and search strategy for the scoping review.

Multimedia Appendix 3 Characteristics of the included studies.

Multimedia Appendix 4 Coding structure of selected data.

## Abbreviations

EHR: electronic health record
EMR: electronic medical record
PHR: personal health record
PRISMA-ScR: Preferred Reporting Items for Systematic Reviews and Meta-Analyses Extension for Scoping Reviews

## Data Availability

All data analyzed during this study are included in this paper.
