# Supplementary material for: 25 Years of Electronic Health Record Implementation Processes: Scoping Review
Source: J Med Internet Res. 2025 Mar 3;27:e60077. doi: 10.2196/60077 (PMC11914847; doi:10.2196/60077)
Supplement: Multimedia Appendix 2 [file jmir_v27i1e60077_app2.docx]

**Multimedia Appendix 2.** Search terms and search strategy for the scoping review.

| Database | Search Terms | Results (n) |
| --- | --- | --- |
| EBSCO (Business Source Complete) | (EHR OR EHRS OR Electronic Health Record* OR EMR OR EMRS OR electronic medical record*) AND implemen* AND process (Abstract)  Filters: 1999-2024, Peer reviewed, full text, academic journals, English | 1238 |
| PubMed | (((((((EHR[Title/Abstract]) OR (EHRS[Title/Abstract])) OR (electronic health record*[Title/Abstract])) OR (EMR[Title/Abstract])) OR (EMRS[Title/Abstract])) OR (electronic medical record*[Title/Abstract])) AND (implemen*[Title/Abstract])) AND (process[Title/Abstract])  Filters: 1999-2024, free full text, English | 1492 |
| Embase | (EHR:ab,ti OR EHRS:ab,ti OR 'electronic health record*':ab,ti OR EMR:ab,ti OR EMRS:ab,ti OR 'electronic medical record*':ab,ti) AND implemen*:ab,ti AND process:ab,ti  Filters: 1999-2024, English, article | 1907 |
| IEEE Explore | ("Abstract":EHR) OR ("Abstract":EHRS) OR ("Abstract":electronic health record*) OR ("Abstract":EMR) OR ("Abstract":EMRS) OR ("Abstract":electronic medical record*) AND ("Abstract":implemen*) AND ("Abstract":process)  Filters: 1999-2024, journals, open access only | 228 |
| Scopus | ( ABS ( EHR ) OR ABS ( EHRS ) OR ABS ( electronic AND health AND record* ) OR ABS ( EMR ) OR ABS ( EMRS ) OR ABS ( electronic AND medical AND record* ) AND ABS ( implemen* ) AND ABS ( process ) )  Filters: 1999-2024, article, English, all open access, journal | 1685 |
|  | **Total** | **6550** |
