# Supplementary material for: 25 Years of Electronic Health Record Implementation Processes: Scoping Review
Source: J Med Internet Res. 2025 Mar 3;27:e60077. doi: 10.2196/60077 (PMC11914847; doi:10.2196/60077)
Supplement: Multimedia Appendix 3 [file jmir_v27i1e60077_app3.docx]

**Multimedia Appendix 3.** Characteristics of the included studies.

| **Author(s)/ Publication year** | **Reference Number** | **Country of Focus** | **Aim** | **Study Design** | **Type of Organization** | **Study Population** | **Record Type** | **Methods** |
| --- | --- | --- | --- | --- | --- | --- | --- | --- |
| Alassia et al. (2017) | [1] | Argentina | Describe a HelpDesk implementation process and its benefits to operational management and excellence applied to the primary care level of Buenos Aires’ public health system. | Qualitative | Primary Care Centers | 44 Primary Care Centers | Electronic Health records (Helpdesk) | Cross Sectional Study. Secondary analysis of Helpdesk’s Database. |
| Banas et al. (2011) | [2] | U.S.A | Provide a case study of the implementation of online provider documentation at a leading medical center with the establishment of an office of clinical transformation (OCT). | Qualitative | Academic Health Centre with an office of clinical transformation | One Academic Health Centre (1451 physicians) | Electronic Health Records | Case Study |
| Barrett and Stephens (2016) | [3] | U.S.A | This study offers a communicative perspective on EHR implementation. Study contributes to AST by empirically demonstrating the pivotal role change appropriation plays in achieving successful change outcomes. This research also contributes to scholarly work on planned organizational change by showing how social interaction, without providing adaptive tools, can create technology implementation problems. | Quantitative | Health care network | Pilot: 12 interviews, 16 online survey. Primary: 340 surveys | Electronic Health Records | Interviews, online survey, data from healthcare network. |
| Bogaert et al. (2021) | [4] | Europe | Identify common enablers and barriers for optimal functioning of HISs across the European Union and associated countries, and to interpret what this means for the further development of HISs in Europe. | Qualitative | National Project | Nine European Countries | Health Information Systems, Electronic Health Records, Population based information systems | Thematic Analysis |
| Boonstra and van Offenbeek (2017) | [5] | The Netherlands | Explore how tendering legislation shapes a buyer's software selection process through the lens of competing decision‐making rationalities. | Qualitative | Large Health Services Provider | One Dutch Large Health Services Provider (31 actors interviewed) | Electronic Health Record | Interpretive single case study, interviews. |
| Boonstra et al. (2017) | [6] | The Netherlands | Analyzes the tensions evolving from project management dilemmas and how they relate to stakeholders in large technology projects | Qualitative | Large Teaching Hospital | 29 interviews | Electronic Health Record | Case Study, interviews, meetings, document review |
| Boswell (2011) | [7] | U.S.A | Explore the perceptions of employees at a multispecialty physician group regarding their readiness to implement HER. | Qualitative | Multi-specialty physician group | 17 offices and 73 providers (16 interviews) | Electronic Health Record | Single Case study, interviews |
| Boswell (2013) | [8] | U.S.A | Report findings on the implementation of electronic health records (EHR) by a multi-specialty physician group in the greater south central Pennsylvania area and offers implications for HR professionals. | Qualitative | Multi-specialty physician group | 16 interviewees. | Electronic Health Record | Case Study, Interviews, |
| Bove et al. (2021) | [9] | U.S.A | Describe the iterative human-centered design and pilot process for multiple sclerosis (MS) NeuroShare, a digital health solution that brings practical information to the point of care so that clinicians and patients with MS can view, discuss, and make informed decisions together | Qualitative | Nonprofit health system | Medical network: 272 primary care clinics, 5,500 physicians, 25 neurology clinics, 62 neurology clinicians) | MS Neuroshare - integrated into EHR. | Human centered design process, interviews, clinic workflow observations and mapping, design sessions, stakeholder advisory group meetings, patient questionnaire. |
| Brokel and Harrison. (2009) | [10] | U.S.A | Describe how Trinity Health (a large multiorganization health care system) addressed EHR-based redesign of care processes in those hospitals preparing to adopt a commercial EHR product, which includes computerized physician order entry (CPOE) and decision support systems (DSS). | Qualitative | Large multiorganization health care system | Fourth largest US Catholic Health Care System: Provides resources to 44 hospitals, 379 outpatient facilities, numerous assisted living, home health, hospice, and senior housing programs through 17 organizations. | Electronic health Records | Case Study |
| Cacciatore et al. (2023) | [11] | Italy | Map the positions of the various actors involved in the realization of EHRs in Italy, focusing on the implementation phase of the NRRP, and comparing the two sub-phases corresponding to the different governments in office, namely, the Draghi government (May 2021 – September 2022) and the Meloni government (since October 2022). | Qualitative | Government | Italian National Resilience and Recovery Plan (Draghi government & Meloni government) | National Electronic Health Record system | Document analysis, literature review, unstructured interviews. |
| Calvo-Amodio et al. (2015) | [12] | U.S.A | An evaluation of the previously-proposed transition phase management model is presented through three different EHR process change case studies. | Quantitative | Two Small Community Health Centers and a large regional hospital | Three case studies | Electronic Health Records | Case Study, historical data, interviews. |
| Carayon et al. (2009) | [13] | U.S.A | Examine the implementation of an electronic health records (EHR) system in a small family practice clinic. Evaluated user experience, work pattern changes, and organizational changes related to the implementation and use of the EHR system. | Mixed Method | Small Family Practice Clinic | Family medicine residency clinic in a small community with a population of about 1800. 6 family medicine faculty, 7 resident physicians, and 1medical support and office staff. Approximately 11 000 patient visits annually. | Electronic Health Record | Survey, interviews, work analysis of staff. |
| Collins et al. (2015) | [14] | U.S.A | To understand existing CI governance structures and provide a model with recommended roles, partnerships, and councils based on perspectives of nursing informatics leaders. | Qualitative | Enterprise wide EHR. | Interview: 12 nursing informatics leaders | electronic health records | Cross sectional study, survey, semi structured interviews. |
| Craven et al. (2014) | [15] | U.S.A | Provide EHR implementation advice for CAHs from a spectrum of experts with an emphasis on recommendations from their peers at CAHs that have undertaken the process. The secondary objective is to begin to identify implementation process differences at CAHs v. larger hospitals. | Qualitative | Critical Access Hospitals | 10 Critical Access Hospitals. Interviews: 41 experts incl. 16 CAH staff members from EHR teams at recently implemented EHRs | Electronic Health Record | Interviews, online survey, data from healthcare network. |
| Cresswell. (2016) | [16] | England | Outline some prevalent existing health IT implementation evaluation frameworks and methods. | Mixed Method | Hospital | Five Hospitals | National EHRs | Case Study, interviews. |
| Crowley et al. (2019) | [17] | U.S.A | Evaluate an electronic health record (EHR) implementation across a large public health department to better understand and improve implementation effectiveness of EHRs in public health departments. | Quantitative | Large suburban county department of health and human services that provides clinical, behavioral, social, and oral health services | 331 staff surveyed prior to EHR implementation, 229 staff surveyed 3 months post EHR implementation | Electronic Health Record | Two phase Cross sectional Survey. |
| Cucciniello et al. (2015) | [18] | Italy | Analyze the coordination practices and mechanisms used for implementing complex innovations in the health care sector, referring specifically to EPRs in two Italian regions, and highlighting any enabling conditions we noted in the two case studies. | Qualitative | Regional Health Care Projects | Lombardy Health Care System: 29 PHs and 15 LHAs. Veneto Health Care System: g twenty-one LHAs and two PHs | Electronic Patient Record | Case Studies, interviews, document analysis. |
| Currie and Finnegan. (2011) | [19] | UK | Reports the findings from a seven-year study on the UK National Health Service on the introduction of an electronic health record for 50 million citizens. | Qualitative | National Project | 123 interviews | Electronic Health Records | Longitudinal research, interviews document analysis. |
| Czerw et al. (2016) | [20] | Poland | Present the starting point, progress, problems and forecasts regarding the implementation of electronic health records at health care entities which provide services within the scope of specialized outpatient care (SOC). | Quantitative | Health Care Entities | 475 health care entities which provide services within the scope of specialized outpatient care (SOC) | Electronic Health Records | Survey Questionnaire |
| Dansky et al. (1999) | [21] | U.S.A | Identify specific attitudes or factors that should be targeted before implementing an EMR project, and demonstrate empirical support for a model of perceived usefulness of EMR.. | Quantitative | Ambulatory care settings | Five private medical practices that are part of a staff plan HMO, and a university based health center. | Electronic Medical Records | Surveys |
| Deokar and Sarnikar (2016) | [22] | U.S.A | Describes how process change issues relate to implementation of large IT projects in healthcare settings. | Qualitative | Hospitals and health systems, physician practices, public health organizations, and community health organizations | Application reports submitted by recipients of the HIMSS Nicholas E. Davies Organizational Award of Excellence during the 10-year period 2000–2010. | Electronic Health Records | Content Analysis |
| deRiel et al. (2018) | [23] | Haiti | To apply Fritz et al.’s framework to a case study of a more mature system: Haiti’s national EMR. | Qualitative | National Project | iSante´, Haiti’s national EMR in use in more than 100 sites and housing records for more than 750 000 patients | National EMR | Case Study, Document analysis. |
| Deutsch et al. (2010) | [24] | England, Germany, Canada, Denmark, Australia | Analyze programs from various countries with regard to the problems documented therein and derive, on a cross-country basis, the most common critical aspects of national electronic health record programs. | Qualitative | National Project | 5 Countries; England, Germany, Canada, Denmark, Australia | Electronic Health Record | Project Reviews and Audits. |
| Evio and Bonito. (2024) | [25] | Philippines | This study determined the factors influencing the implementation of eHealth solutions in the Philippines, in consideration of the development process and initial outputs of the Philippine eHealth Strategic Framework and Plan 2014-2020. | Qualitative | National Project | 15 municipalities/cities in the Philippines | eHealth (electronic health records and patient registries) | Records review, interviews, content analysis. |
| Faiella et al. (2019) | [26] | U.S.A | Describe the implementation of enhanced health information technology (HIT), specifically an electronic health record (EHR), into the workflow of a charitable community pharmacy and to highlight the impact of the EHR on clinical service advancement, student and resident learning, research, and grant support for the pharmacy. | Qualitative | Nonprofit Community Pharmacy | The Charitable Pharmacy of Central Ohio has served over 6700 unique patients. The pharmacy is staffed by 1 full-time and 2 part-time pharmacists, 2 pharmacy technicians, 2 PGY-1 community pharmacy residents, and 4-5 Advanced Pharmacy Practice Experience (APPE) students. | cloud based Electronic Health Record | Feedback from stakeholders, discussion at staff meetings, quality improvement project. |
| Felt-Lisk et al. (2010) | [27] | U.S.A | Offer current insights into the barriers and facilitators of EHR use in small and medium-sized practices. | Qualitative | small to medium sized physician practices | 32 small- to medium sized physician practices in four States | Electronic Health Record | Interviews, online survey, data from healthcare network. |
| Fleming et al. (2011) | [28] | U.S.A | Inform real world health IT implementation decisions, especially in the context of the current national priority placed on the adoption of EHRs. Stimulate more comprehensive research on health IT implementation in the ambulatory care setting by clarifying the costs related to the implementation of f an electronic health record system. | Mixed Method | Single large physician network | 26 primary care practices in a physician network | Electronic Health Record | Interviews, cost data analysis. |
| Ford et al. (2010) | [29] | U.S.A | Assess complete versus incomplete HIT implementation levels among US hospitals in light of the various technology adoption strategies employed. Discuss the implications with respect to meaningful use for hospitals that have adopted the different HIT strategies. | Quantitative | Hospitals | 1814 hospitals (12.7 percent for profit) (59.2 percent members of a hospital system) | Electronic Health Records | Logistic regression, Survey. |
| Fragidis and Chatzoglou. (2018) | [30] | Denmark, Austria, Sweden, Norway, the UK, Germany, the Netherlands, Switzerland, Canada, the USA, Israel, New Zealand and South Korea | Identify the best practices applied during the implementation process of a national electronic health record (EHR) system. Explore the knowledge gained by experts from leading countries in the field of nationwide EHR system implementation. | Quantitative | National health systems | 13 countries | Electronic Health Record | Survey |
| Gabriel et al. (2014) | [31] | U.S.A | Examine electronic health record (EHR) adoption, key EHR functionalities, telehealth, and teleradiology, as well as challenges to EHR adoption. | Quantitative | Critical Access Hospitals | 793 hospitals | Electronic Health Record | Survey |
| Gans et al. (2005) | [32] | U.S.A | Assess current use of IT in medical group practices. | Quantitative | Medical Group Practices | Random sample of34,490 medical groups | Electronic Health Record | Web, mail, telephone surveys. |
| Garrety et al. (2016) | [33] | Australia | Show how these projects can ironically take on the characteristics of the ‘wicked problems’ they are intended to solve, and how a failure to recognize and cope with these ‘wicked’ characteristics can lead to waste, conflict and frustration among potential users. | Qualitative | National Project | Interviews: 13 people | National Electronic Health Record | Case Study, document analysis, interviews. |
| Ghani et al. (2008) | [34] | Malaysia, Singapore, Japan, Hong Kong, Taiwan | Presents an overview of the development approaches undertaken by four East Asian countries in implementing a national Electronic Health Record (EHR) in the public health system. | Qualitative | National Project | Four East Asian Countries | National Electronic Health Record (EHR) / Lifetime Health Record (LHR) | Literature Search, case study, interviews, archival documents. |
| Greenhalgh et al. (2008) | [35] | England | Explore the introduction of a centrally stored, shared electronic patient record (the summary care record (SCR)) in England and draw wider lessons about the implementation of large scale information technology projects in health care. | Mixed Method | Four early adopter sites (Each site: f a primary care trust, participating general practices, and one or more linked unscheduled care setting(such as an emergency department, walk-in center, out of hours service). | 250 staff interviews, 1500 hours of ethnographic observation, interviews and focus groups with 170 patients and carers, 2500 pages of correspondence and documentary evidence, and incorporation of relevant surveys and statistics produced by others | Shared Electronic Records | Interviews, document analysis, surveys. |
| Greenhalgh et al. (2010) | [36] | England | Evaluate the policy making process, implementation by NHS organizations, and patients’ and carers’ experiences of efforts to introduce an internet accessible personal electronic health record (Health Space) in a public sector healthcare system. | Mixed method | Website | s 56 patients and carers, 3000 pages of documents, 160 interviews with policy makers, project managers, and clinical staff. | Personal Electronic Health Record | Multilevel case study. |
| Gross et al. (2016) | [37] | U.S.A | Describes how trust among team members and in the technology supporting them was eroded during implementation of an electronic health record (EHR) in an adult outpatient oncology practice at a comprehensive cancer center. | Qualitative | Comprehensive cancer center | One adult outpatient oncology practice | Electronic Health Record | Case study |
| Hariyati et al. (2020) | [38] | Indonesia | Explore and describe the usability and satisfaction of using electronic documentation. | Mixed Method | Hospitals | Focus group interviews involving eight nurses and a questionnaire survey to 219 nurses. | Electronic Nursing Documentation | Focus group interviews and a questionnaire survey. |
| Heath and Porter. (2018) | [39] | U.S.A | Gain understanding into the human factors which might impede the change process. | Qualitative | Hospitals | 28 physicians interviewed | Electronic Health Record | Semi structured interviews |
| Heisey-Grove et al. (2014) | [40] | U.S.A | Summarizes challenges to EHR adoption and  MU based on nationwide data supplied by 55 Regional Extension  Centers reporting over 19,000 issues representing over 43,000 unique  health care providers. | Quantitative | Regional Extension Centers | 55 Regional Extension Centers | Electronic Health record | Analysis of challenge reports. |
| Helton et al. (2017) | [41] | U.S.A | Study how decision makers in practices can view their available choices when implementing EHR software. | Qualitative | Small Clinics | Dental practice, Nonprofit health care organization, Pulmonary/family practice. | Electronic Health Records | Multi Case Study, interviews. |
| Heponiemi et al. (2021) | [42] | Finland | Examine the associations of EHR-to-EHR implementations and the sufficiency of related training with perceived stress related to information systems (SRIS), time pressure, and cognitive failures among registered nurses. Moreover, we examined the moderating effect of the employment sector (hospital, primary care, social services, and others) on these associations. | Quantitative | Hospitals | 3610 registered Finnish nurses | Electronic Health Records | Cross sectional survey |
| Hernández-Ávila et al. (2013) | [43] | Mexico | Assessing the design and implementation of an electronic health record (EHR) in the public health system of Colima, Mexico. | Qualitative | 2 health centers and 2 hospitals | 27 interviews, 4 focus groups | Electronic Health Record | Interviews and focus group discussions. |
| Hertzum and Ellingsen. (2019) | [44] | Norway, UK, Denmark | Compare the experiences from implementing Epic in the UK and Denmark with the preparations for implementing it in Norway. | Qualitative | National Projects | 6 interviews | Electronic Health Records | Document analysis and interviews. |
| Hertzum et al. (2021) | [45] | Norway | Investigate how the early implementation process drives their expectations of an EHR that is being implemented in Norway. | Qualitative | GP clinics | 9 interviewees | Electronic Health Records | Interviews |
| Hertzum et al. (2022) | [46] | Denmark and Finland | Analyze the Epic implementations in Denmark and Finland to understand how healthcare professionals experience this large-scale HER. | Quantitative | National Project | Document Analysis | Electronic Health Records | Documentary analysis, user surveys, assessment reports. |
| Jung et al. (2020) | [47] | Russian Far East | Qualitatively investigate and analyze the current status of EHRs in the Russian Far East and derive implementation plans for nationwide EHRs. | Qualitative | State medical information center, national children’s hospital, outpatient hospital, private children’s hospital, federal university hospital, Department of Health | 25 interviewees | Electronic Health Record | Semi Structured interviews |
| Kang'a et al. (2017) | [48] | Kenya | Focuses on the specific activities undertaken by I-TECH in Kenya in support of the Ministry of Health in Kenya focusing on the people, processes and technologies employed in the EMR implementation. | Qualitative | Health Facilities | 342 implementations | Electronic Medical Records | Monitoring and evaluation data. |
| Kiepek and Sengstack. (2019) | [49] | U.S.A | Evaluate end-user support processes and personnel employed during the initial phase of EHR implementation at an academic medical center and identify facilitators of success, challenges, and lessons learned. | Qualitative | Large, complex health care system | One Complex Health Care System - main campus comprised an adult hospital, a children’s hospital, a behavioral health hospital, and a rehabilitation hospital with 1,131 licensed beds in total, ambulatory clinics located on campus and in over 240 remote locations | Electronic Health Records | Case Study |
| Klecun et al. (2019) | [50] | Singapore, England | Proposes a framework that draws on both stakeholder and institutional theories to understand the complex dynamics of stakeholder interactions and institutional pressures over time during electronic health record systems implementation. | Qualitative | National Project | Singapore: 15 interviews, 74 documents. England (33 interviews previously conducted) | Electronic Health records | Interpretive case study, interviews, document analysis. |
| Knight et al. (2014) | [51] | Australia | The National E-health Transition Authority contracted the Improvement Foundation Australia to conduct a quality improvement collaborative based on 9 years of experience with the Australian Primary Care Collaborative Program. | Qualitative | National Project | 56 practices. 926 patients. 650 shared health summaries uploaded. 519 patient views. 421 plan/do/study/acts submitted | Patient-controlled electronic health record | Workshops, reviewing data from healthcare summaries. |
| Maier et al. (2022) | [52] | Denmark | Study the development of a smoldering crisis over time. The focus is on a nationwide news media and online news communication related to a smoldering crisis running in the Danish healthcare system since 2016: the problematic implementation of a large-scale electronic health record (EHR), technology entitled Sundhedsplatformen (SP), in the hospitals of the capital region of Denmark. | Qualitative | National Project | 84 News Articles | Electronic Health Record | Case Study, longitudinal investigation of online news articles. |
| Marca et al. (2014) | [53] | Spain | Describe the level of adoption of electronic health records in Spanish hospitals and to identify potential barriers and facilitators to this process. | Quantitative | Hospitals | 64 survey responses | Electronic Health Records | Observational cross-sectional design, survey. |
| Martin et al. (2022) | [54] | U.S.A | This exploratory case study looked to access socio-technical barriers and facilitators to EHR implementation specifically in the military. | Qualitative | Military Health System | implementation plans, evaluation reports, congressional reports, news articles, and relevant peer-reviewed literature related to military EHR implementation. | Electronic Health Record | Exploratory case study, Document review. |
| Mbwambo and Mandari. (2023) | [55] | Tanzania | Assessed factors that influence the acceptance of interoperable electronic Health Records (EHRs) Systems in Tanzania Public Hospitals. | Quantitative | Clinics, polyclinics and hospitals | 281 responses | Electronic Health Records | Questionnaire |
| McAlearney et al. (2013) | [56] | U.S.A | To characterize elements of successful electronic health record (EHR) system implementation and to synthesize the key informants’ perspectives about successful implementation practices. | Qualitative | Exemplars for successful ambulatory EHR implementation. | 45 physician interviews (six organizations) 6 focus groups (37 physician providers) | Electronic Health Records | Interviews, focus groups. |
| McAlearney et al. (2014) | [57] | U.S.A | Comprehensively study and synthesize best practices for managing ambulatory EHR system implementation in healthcare organizations, highlighting applicable management theories and successful strategies. | Qualitative | Health care organization | 45 Interviews (in healthcare organizations. 6 focus groups (with 37 physicians) | Electronic Health Records | Interviews, focus groups. |
| McGinn et al. (2012) | [58] | Canada | Understanding EHR users’ perspectives is key to the success of EHR implementation projects. This Delphi study aimed to assess in the Canadian context the applicability, the importance, and the priority of pre-identified factors from a previous mixed-methods systematic review of international literature. | Quantitative | Healthcare professional associations, organizations and interest groups | 64 participants from 4 EHR user groups (non-physician healthcare professionals, health information professionals, managers, and physicians.) | Electronic Health Records | Delphi Study |
| Muinga et al. (2018) | [59] | Kenya | Present a descriptive case study of the implementation of an open source electronic health record system in public health care facilities in Kenya. | Qualitative | Public Hospitals | 5 site visits | Open Source Electronic Health Records | Historical case study, Semi structured interviews, group discussions. |
| Naeem and Alqasumi. (2020) | [60] | Kingdom of Saudia Arabia | Uncover and address the issues of electronic medical record (EMR) implementation in public sector hospitals in the Kingdom of Saudi Arabia (KSA). | Qualitative | Public Sector Hospitals | 40 participants | Electronic Medical Records | Interviews |
| Noblin et al. (2013) | [61] | U.S.A | Prior studies focused on conversions from paper to electronic records. Many provider impressions, therefore, may have been influenced by reactions to the process of being required to change well established patterns. In order to help separate such reactions from true evaluations of the efficacy of the EHR, we decided to survey the providers in a new health center. | Qualitative | New health center | 7 interviews (3 physicians, 4 medical assistants) | Electronic Health Records | Case study, survey, semi structured interviews. |
| Palvia et al. (2015) | [62] | U.S.A | Using the lens of stakeholder theory, we examine the differing views of stakeholders (namely, medical providers and vendors) in the implementation of electronic health record (EHR) systems. | Quantitative | Clinical Providers and Vendors | Pilot study (13 executives from EHR vendors) Full Study (328 responses) | Electronic Health Records | Surveys (based on EHR readiness surveys). |
| Pearce et al. (2014) | [63] | Australia | Describes the processes undertaken and the experiences of introducing the PCEHR into 74 general practices across a specific area of metropolitan Melbourne. | Quantitative | General Practices | 84 staff responses | Personally Controlled Electronic Health Record | Online survey |
| Pine et al. (2016) | [64] | U.S.A | Empirically examine how different forms of coordinating emerge depending on the ways in which integrating conditions are achieved in practice. | Qualitative | Mid-sized independent university-owned teaching hospital | 55 observations (varying in time from 4-14 hours) | Electronic Health records | Participant observation over 16 months. 32 semi structured interviews (32 caregivers, 28 additional). |
| Pohlmann et al. (2020) | [65] | Germany | Identify policies, structures, and practices of the German health care system that influence the uptake and use of a PHR. | Qualitative | National Project | 33 interviews (23 different health care professionals, 10 key actors in German health care system eHealth experts) | Personal Electronic Health Record | semi structured interviews |
| Poss-Doering et al. (2018) | [66] | Germany | Outline findings of the posttrial qualitative study carried out to evaluate user-reported experiences, perceptions, and perspectives, focusing on their interpretation of PEPA beyond technical usability and views on a future nationwide implementation | Qualitative | General Practice | Interviews (11 patients, 3 physicians) | Web-based personal electronic health record prototype | Semi structured guide based interviews. |
| Rau et al. (2024) | [67] | Germany | characterize the structural factors relating to the adoption of the EHR in more detail from the perspective of representatives of stakeholders working in the German healthcare system and to identify existing barriers to implementation and the need for change. | Qualitative | health insurance, pharmacies, healthcare research, EHR development and panel doctors | 5 male interviewees | Electronic Health Record | Expert interviews |
| Robertson et al. (2010) | [68] | England | Describe and evaluate the implementation and adoption of detailed electronic health records in secondary care in England and thereby provide early feedback for the ongoing local and national rollout of the NHS Care Records Service. | Mixed Method | Acute hospital and mental health trusts | 5 acute hospitals and mental health trusts | Electronic Health Records | longitudinal, multisite, sociotechnical case study, semi structured interviews, document analysis, field notes, quant data. |
| Scott et al. (2005) | [69] | Hawaii | Examine users’ attitudes to implementation of an electronic medical record system in Kaiser Permanente Hawaii. | Qualitative | Clinics, Hospitals | 4 primary healthcare teams in four clinics & 4 specialty departments in one hospital. (26 senior clinicians, managers , and project team members) | Electronic Medical Records | Semi structured Interviews |
| Ser et al. (2014) | [70] | England | Investigate the perceptions and reported practices of mental health hospital staff using national hospital electronic health records (EHRs) in order to inform future implementations, particularly in acute mental health settings. | Qualitative | Mental Health Hospitals | 33 interviews | Electronic Health Records | Secondary analysis of semi structured interview data. |
| Sheehan et al. (2023) | [71] | Ireland | Describe the key observations and lessons learned from the national project team implementing the MN-CMS. | Qualitative | Maternity Units/ Hospitals | National Project | Electronic Health Records | Discussions with the project team, post go-live workshops, phase one closure report developed by the national project team in conjunction with key stakeholders. |
| Sheikh et al. (2011) | [72] | England | Evaluate the implementation and adoption of the NHS detailed care records service in “early adopter” hospitals in England | Qualitative | Hospitals | 431 interviews, 590 hours of observations | Electronic health records | Interviews, observations, document analysis. |
| Shield et al. (2010) | [73] | U.S.A | Examine the effects of EHR implementation, especially regarding physician-patient communication and behaviors and patients’ responses. | Mixed Method | Family Medicine outpatient center | 170 clinical encounters | Electronic Health Record | 22-month, triangulation design, interviews, time measurements of clinical encounters, focus groups, unstructured observations. |
| Sidek and Martins. (2017) | [74] | Brunei | Identify the perceived critical success factors of EHR system implementation in a dental clinic context. | Qualitative | Dental Clinic | 11 interviews | Electronic Health Record | Case study, Grounded theory, interviews, focus groups. |
| Snowden and Kolb. (2017) | [75] | Scotland | Explore the impact of implementing an electronic health record system on staff at a Scottish hospice. | Mixed Method | Hospice | 150 employees of hospice | Electronic Health Record | Surveys, focus groups. |
| Stanczyk et al. (2017) | [76] | Netherlands | Assess the role of involvement and its effects on socio-cognitive beliefs regarding the implementation of a new EHR system. | Quantitative | Hospital | 359 questionnaire responses | Electronic Health Records | Questionnaire |
| Standing and Cripps. (2013) | [77] | Slovenia, Australia | Identify the factors impacting on successful e-health implementation. | Qualitative | National Project | Australia (23 participants) Slovenia (19 interviews) | Electronic Health Records | Case studies, interviews. |
| Strong et al. (2014) | [78] | U.S.A | identify points of leverage for managers in order to improve the record of reaching desired goals from EHR investments. | Qualitative | Multisite medical group | 110 interviews | Electronic Health Record | Longitudinal study, grounded theory, interviews. |
| Takian et al. (2012) | [79] | England | Describes the arrival, the process of implementation, stakeholders’ experiences and the local consequences of the implementation of an EHR system into a mental health hospital. | Qualitative | Mental Health Hospital | 48 interviews, 26 hours observations, 65 documents | Electronic Health Record | Longitudinal, case study, interviews, document analysis. |
| Takian et al. (2014) | [80] | England | Explore the role of organizational learning in enabling implementation and supporting adoption of electronic health record systems into two English hospitals. | Qualitative | Hospitals | 63 interviews, 41 hours observations, 218 documents | Electronic Health Records | Longitudinal, case study, interviews, document analysis. |
| Takian. (2012) | [81] | England | Reports the arrival, implementation process, and stakeholders’ experiences of one EHR software (Millennium) at a National Health Service’s (NHS) general hospital participating in NPfIT. | Qualitative | Hospital | 63 interviews, 22 hours of observation, 123 documents | Electronic Health Record | Interviews, documentary analysis, observation. |
| Threatt et al. (2019) | [82] | U.S.A | Describe the design objectives, capabilities, adoption, and provide usage statistics of a mobile application that assisted in our largescale EHR implementation and change management process. | Mixed Method | University Medical Centre | 727 task logs | Electronic Health Records | Agile process design. |
| Tobler et al. (2016) | [83] | U.S.A | Explains CMUA, prior research that uses the CMUA model, and the need to study over time and within time. | Mixed Method | Ambulatory, multispecialty group | Seven clinicians, five mid-level practitioners, 25 clinical staff, and 15 administrative staff (15 clinicians observed) | Electronic Health Records | Quasi experimental, observation. |
| Trocin et al.(2024) | [84] | Italy | Advance our knowledge of the emergence of unintended consequences from the implementation of Electronic Health Record (EHR) systems. | Qualitative | Primary and secondary care | Region Project (31 interview participants) | Electronic Health Records | Case Study, semi structured interviews. |
| Vadillo et al. (2016) | [85] | U.S.A | Explored factors influencing EHR adoption in a critical care unit (CCU) at a small urban hospital and ways to better engage staff in the process. | Mixed Method | Small Urban Hospital | 65 (survey) 6 (focus group) | Electronic Health records | Survey, focus group. |
| van Offenbeek and Vos. (2015) | [86] | The Netherlands | Develops a multilayered stakeholder–issue framework that makes the connections between stakeholders and issues explicit with the aim of helping project managers analyze and prioritize the issues that stakeholders confront them with. | Mixed Method | Large Teaching Hospital | 583 (survey) 13 (interviews) | Electronic Health Record | Design Approach, interviews, survey. |
| van Offenbeek et al. (2023) | [87] | Denmark | Unravel how clinical departments’ adoption of this organization-wide system was primarily shaped by their critical work system dependencies rather than their role during its implementation. | Qualitative | large hospital | 36 interviews | Electronic Health Records | Interpretive case study, interviews. |
| Weston et al. (2023) | [88] | U.S.A | Describe the first year of implementation of the integrated health care delivery model, barriers to implementation, challenges to sustainability, and successes. | Qualitative | Clinics | 5 clinics | Electronic Health Records | Case study, interviews, document analysis, observations. |
| Yung. (2017) | [89] | Australia | Present details of the adoption process, use of the EHR system, physiotherapists’ satisfaction and concerns. | Qualitative | Physiotherapy Clinic | 2 clinics | Electronic Health Record | Case Study, observation, demi structured interviews. |
| Zandieh et al. (2008) | [90] | U.S.A | Determine how ambulatory leaders differentiate implementation approaches between practices that are currently paper-based and those with a legacy EHR system (EHR-based). | Qualitative | Large Teaching Hospital | 23 interviewees. | Electronic Health Record | Interviews |

## References

1. Alassia L, Palermo C, Recondo F, Giussi M, Stieben A, Baum A, Gonzalez Bernaldo de Quiros F. Managing user needs during the EHR implementation in Buenos Aires City: The HelpDesk role. *Precision Healthcare Through Informatics.* 2017:835-839. doi:10.3233/978-1-61499-830-3-835.
2. Banas CA, Ewing SK, Tarczy-Hornoch P, et al. Phased implementation of electronic health records through an office of clinical transformation*. J Am Med Inform Assoc.* 2011;18(5):721-725. doi:10.1136/amiajnl-2011-000165.
3. Barrett AK, Stephens KK. The pivotal role of change appropriation in the implementation of health care technology. *Manage Commun Q*. 2016;31(2):163-193. doi:10.1177/0893318916682872.
4. Bogaert P, Van Oyen H, Silviu R, et al. Identifying common enablers and barriers in European health information systems*. Health Policy*. 2021;125(12):1517-1526. doi:10.1016/j.healthpol.2021.09.006.
5. Boonstra A, van Offenbeek M. Shaping a buyer’s software selection process through tendering legislation. *Information Systems Journal*. 2018;28(5):905-928. doi:10.1111/isj.12174.
6. Boonstra A, Koutsikouri D, Offenbeek MAGV. Tension awareness of stakeholders in large technology projects: A duality perspective*. Proj Manag J*. 2017;48(1):19-36. doi:10.1177/875697281704800102.
7. Boswell RA. A physician group’s movement toward electronic health records: a case study using the transtheoretical model for organizational change*. Consult Psychol J Pract Res.* 2011;63(2):138-148. doi:10.1037/a0024319.
8. Boswell RA. Implementing electronic health records: implications for HR professionals*. Strateg HR Rev.* 2013;12(5):262-268. doi:10.1108/shr-08-2012-0010.
9. Bove R, Rhodes J, Shiner CT, et al. Electronic health record technology designed for the clinical encounter*. Neurol Clin Pract.* 2021;11(4):318-326. doi:10.1212/cpj.0000000000000986.
10. Brokel JM, Harrison MI. Redesigning care processes using an electronic health record: a system’s experience. *Jt Comm J Qual Patient Saf*. 2009;35(2):82-92. doi:10.1016/s1553-7250(09)35011-4.
11. Cacciatore F, Natalini A, Saruis T. Interest groups and the implementation of electronic health records in the Italian NRRP, between policy and politics. *Contemp Ital Polit.* 2023;16(1):21-38. doi:10.1080/23248823.2023.2288976.
12. Calvo-Amodio J, Patterson PE, Smith AE, Fawcett SA. Application of transition-phase management model for an electronic health record system implementation: a case study*. Eng Manag J*. 2015;27(3):131-140. doi:10.1080/10429247.2015.1064662.
13. Carayon P, Smith P, Hundt AS, Kuruchittham V, Li Q. Implementation of an electronic health records system in a small clinic: the viewpoint of clinic staff. *Behav Inf Technol.* 2009;28(1):5-20. doi:10.1080/01449290701628178.
14. Collins SA, Alexander D, Moss J, et al. Nursing domain of CI governance: recommendations for health IT adoption and optimization. *J Am Med Inform Assoc*. 2015;22(3):697-706. doi:10.1093/jamia/ocu001.
15. Craven CK, Sievert MC, Hicks LL, Alexander GL, Hearne LB, Holmes JH. CAH to CAH. *Appl Clin Inform.* 2014;5(1):92-117. doi:10.4338/ACI-2013-08-RA-0066.
16. Cresswell K. Evaluation of implementation of health IT. *Stud Health Technol Inform*. 2016;222:206-219. PMID: 27198104.
17. Crowley K, Mishra A, Cruz-Cano R, Gold R, Kleinman D, Agarwal R. Electronic health record implementation findings at a large, suburban health and human services department*. J Public Health Manag Pract*. 2019;25(1). doi:10.1097/PHH.0000000000000768. PMID: 29324567; PMCID: PMC7329137.
18. Cucciniello M, Guerrazzi Young C, Nasi G, Ongaro E. Coordination mechanisms for implementing complex innovations in the health care sector. *Public Manag Rev.* 2015;17:1-21. doi:10.1080/14719037.2015.1029348.
19. Currie W, Finnegan DJ. The policy-practice nexus of electronic health records adoption in the UK NHS. *J Enterp Inf Manag*. 2011;24:146-170. doi:10.1108/17410391111106284.
20. Czerw A, Fronczak A, Witczak K, Juszczyk G. Implementation of electronic health records in Polish outpatient health care clinics: starting point, progress, problems, and forecasts. *Ann Agric Environ Med*. 2016;23(2):329-334. doi:10.5604/12321966.1203900. PMID: 27294642.
21. Dansky KH, Gamm LD, Vasey JJ, Barsukiewicz CK. Electronic medical records: are physicians ready? *J Healthc Manag*. 1999;44(6):440-454; discussion 454-455. PMID: 10662431.
22. Deokar A, Sarnikar S. Understanding process change management in electronic health record implementations*. Inf Syst e-Bus Manage.* 2016;14:733-766. doi:10.1007/s10257-014-0250-7.
23. deRiel E, Puttkammer N, Hyppolite N, et al. Success factors for implementing and sustaining a mature electronic medical record in a low-resource setting: a case study of iSanté in Haiti. *Health Policy Plan*. 2018;33(2):237-246. doi:10.1093/heapol/czx171. PMID: 29253138.
24. Deutsch E, Duftschmid G, Dorda W. Critical areas of national electronic health record programs—Is our focus correct? *Int J Med Inform*. 2010;79(3):211-222. doi:10.1016/j.ijmedinf.2009.12.002. PMID: 20079685.
25. Evio BD, Bonito SR. Formative evaluation of the implementation of eHealth in the Philippines: a qualitative study*. Acta Med Philipp*. 2024;58(12):35-47. doi:10.47895/amp.v58i12.9289. PMID: 39071527; PMCID: PMC11272894.
26. Faiella A, Casper KA, Bible L, Seifert J. Implementation and use of an electronic health record in a charitable community pharmacy. *J Am Pharm Assoc* (2003). 2019;59(2S). doi:10.1016/j.japh.2018.12.004. PMID: 30733152.
27. Felt-Lisk S, Johnson L, Fleming C, Shapiro R, Natzke B. Toward understanding EHR use in small physician practices*. Health Care Financ Rev*. 2010;31(1):11-22. PMID: 20191754; PMCID: PMC4195064.
28. Fleming NS, Culler SD, McCorkle R, Becker ER, Ballard DJ. The financial and nonfinancial costs of implementing electronic health records in primary care practices. *Health Aff (Millwood).* 2011;30(3):481-489. doi:10.1377/hlthaff.2010.0768. PMID: 21383367.
29. Ford EW, Menachemi N, Huerta TR, Yu F. Hospital IT adoption strategies associated with implementation success: implications for achieving meaningful use. *J Healthc Manag.* 2010;55(3):175-188; discussion 188-189. PMID: 20565034.
30. Fragidis LL, Chatzoglou PD. Implementation of a nationwide electronic health record (EHR). *Int J Health Care Qual Assur.* 2018;31(2):116-130. doi:10.1108/IJHCQA-09-2016-0136. PMID: 29504871.
31. Gabriel MH, Jones EB, Samy L, King J. Progress and challenges: implementation and use of health information technology among critical-access hospitals. *Health Aff (Millwood).* 2014;33(7):1262-1270. doi:10.1377/hlthaff.2014.0279. PMID: 25006155.
32. Gans D, Kralewski J, Hammons T, Dowd B. Medical groups' adoption of electronic health records and information systems. *Health Aff (Millwood).* 2005;24(5):1323-1333. doi:10.1377/hlthaff.24.5.1323. PMID: 16162580.
33. Garrety K, McLoughlin I, Dalley A, Wilson R, Yu P. National electronic health record systems as ‘wicked projects’: The Australian experience. *Inf Polity*. 2016;21(4):367-381. doi:10.3233/ip-160389.
34. Ghani MKA, Bali RK, Naguib RNG, Marshall IM, Wickramasinghe NS. Electronic health records approaches and challenges: a comparison between Malaysia and four East Asian countries. *International Journal of Electronic Healthcare*. 2008;4(1):78. doi:10.1504/ijeh.2008.018922.
35. Greenhalgh T, Stramer K, Bratan T, Byrne E, Mohammad Y, Russell J. Introduction of shared electronic records: multi-site case study using diffusion of innovation theory. *BMJ.* 2008;337. doi:10.1136/bmj.a1786. PMID: 18948344; PMCID: PMC3269664.
36. Greenhalgh T, Hinder S, Stramer K, Bratan T, Russell J. Adoption, non-adoption, and abandonment of a personal electronic health record: case study of HealthSpace. *BMJ.* 2010;341. doi:10.1136/bmj.c5814. PMID: 21081595; PMCID: PMC2982892.
37. Gross AH, Leib RK, Tonachel A, Tonachel R, Bowers DM, Burnard RA, Rhinehart CA, Valentim R, Bunnell CA. Teamwork and electronic health record implementation: a case study of preserving effective communication and mutual trust in a changing environment. *J Oncol Pract*. 2016;12(11):1075-1083. doi:10.1200/JOP.2016.013649. PMID: 27601513.
38. Hariyati Rr, Hamid AY, Eryando T, Hasibuan Z. Usability and satisfaction of using electronic nursing documentation: lesson-learned from new system implementation at a hospital in Indonesia*. Int J Healthc Manage*. 2020;13:1-8. doi:10.1080/20479700.2018.1504387.
39. Heath M, Porter T. Change management overlooked: physician perspectives on EHR implementation. Am J Bus. 2018;34. doi:10.1108/AJB-09-2017-0028.
40. Heisey-Grove D, Danehy LN, Consolazio M, Lynch K, Mostashari F. A national study of challenges to electronic health record adoption and meaningful use. *Med Care.* 2014;52(2):144-148. doi:10.1097/MLR.0000000000000038. PMID: 24309669.
41. Helton J, Wade D, Erhardt R. Small clinic electronic health records implementations: an options thinking view. *J Manag Policy Pract.* 2017;18(1):28-43.
42. Heponiemi T, Gluschkoff K, Vehko T, et al. Electronic health record implementations and insufficient training endanger nurses' well-being: cross-sectional survey study. *J Med Internet Res*. 2021;23(12). doi:10.2196/27096. PMID: 34941546; PMCID: PMC8738988.
43. Hernández-Ávila JE, Palacio-Mejía LS, Lara-Esqueda A, Silvestre E, Agudelo-Botero M, Diana ML, Hotchkiss DR, Plaza B, Sanchez Parbul A. Assessing the process of designing and implementing electronic health records in a statewide public health system: the case of Colima, Mexico*. J Am Med Inform Assoc*. 2013;20(2):238-244. doi:10.1136/amiajnl-2012-000907. Epub 2012 Sep 27. PMID: 23019239; PMCID: PMC3638180.
44. Hertzum M, Ellingsen G. The implementation of an electronic health record: comparing preparations for Epic in Norway with experiences from the UK and Denmark. *Int J Med Inform*. 2019;129:312-317. doi:10.1016/j.ijmedinf.2019.06.026. Epub 2019 Jun 26. PMID: 31445272.
45. Hertzum M, Ellingsen G, Melby L. Drivers of expectations: why are Norwegian general practitioners skeptical of a prospective electronic health record? *Health Inform J*. 2021;27(1). doi:10.1177/1460458220987298.
46. Hertzum M, Ellingsen G, Cajander Å. Implementing large-scale electronic health records: experiences from implementations of Epic in Denmark and Finland*. Int J Med Inform.* 2022;167:104868. doi:10.1016/j.ijmedinf.2022.104868. Epub 2022 Sep 14. PMID: 36194994.
47. Jung SY, Lee K, Lee HY, Hwang H. Barriers and facilitators to implementation of nationwide electronic health records in the Russian Far East: A qualitative analysis. *Int J Med Inform*. 2020;143:104244. doi:10.1016/j.ijmedinf.2020.104244.
48. Kang'a SG, Muthee VM, Liku N, Too D, Puttkammer N. People, Process and Technology: Strategies for Assuring Sustainable Implementation of EMRs at Public-Sector Health Facilities in Kenya. *AMIA Annu Symp Proc.* 2017;2016:677-685. Published 2017 Feb 10.
49. Kiepek W, Sengstack PP. An Evaluation of System End-User Support during Implementation of an Electronic Health Record Using the Model for Improvement Framework. *Appl Clin Inform*. 2019;10(5):964-971. doi:10.1055/s-0039-3402450.
50. Klecun E, Zhou Y, Kankanhalli A, Wee Y, Hibberd R. The dynamics of institutional pressures and stakeholder behavior in national electronic health record implementations: a tale of two countries. *J Inform Technol.* 2019;34:026839621882247. doi:10.1177/0268396218822478.
51. Knight AW, Szucs C, Dhillon M, Lembke T, Mitchell C. The eCollaborative: using a quality improvement collaborative to implement the National eHealth Record System in Australian primary care practices. *Int J Qual Health Care.* 2014 Aug;26(4):411-7. doi: 10.1093/intqhc/mzu059. Epub 2014 Jun 12. PMID: 24925685; PMCID: PMC4126615.
52. Maier C, Frandsen F, Johansen W. Understanding the arena of smoldering crises: a longitudinal study of discursive struggles after implementing a new IT health care platform. *J Commun Manag*. 2022;27. doi:10.1108/JCOM-12-2021-0136.
53. Marca G, Perez A, Blanco-Garcia MG, Miravalles E, Soley P, Ortiga B. The use of electronic health records in Spanish hospitals. *Health Inf Manag.* 2014;43(3):37-44. doi:10.1177/183335831404300305.
54. Martin MB, Petros M, Welter C. Exploratory Case Study of Barriers and Facilitators Associated With the Pilot Implementation of a New Electronic Healthcare Record in the Military. Mil Med. 2022;187(3-4):e486-e492. doi:10.1093/milmed/usab053.
55. Mbwambo E, Mandari H. Acceptance of interoperable electronic health record (EHRs) systems: a Tanzanian e-health perspective. *J Int Technol Inf Manag.* 2023;32(1) 5. doi:10.58729/1941-6679.1574.
56. McAlearney AS, Sieck C, Hefner J, Robbins J, Huerta TR. Facilitating ambulatory electronic health record system implementation: evidence from a qualitative study. *Biomed Res Int*. 2013;2013:629574. doi:10.1155/2013/629574.
57. McAlearney AS, Hefner JL, Sieck C, Rizer M, Huerta TR. Evidence-based management of ambulatory electronic health record system implementation: an assessment of conceptual support and qualitative evidence*. Int J Med Inform.* 2014;83(7):484-494. doi:10.1016/j.ijmedinf.2014.04.002.
58. McGinn CA, Gagnon MP, Shaw N, Sicotte C, Mathieu L, Leduc Y, Grenier S, Duplantie J, Abdeljelil AB, Légaré F. Users' perspectives of key factors to implementing electronic health records in Canada: a Delphi study. *BMC Med Inform* Decis Mak. 2012 Sep 11;12:105. doi: 10.1186/1472-6947-12-105. PMID: 22967231; PMCID: PMC3470948.
59. Muinga N, Magare S, Monda J, et al. Implementing an Open Source Electronic Health Record System in Kenyan Health Care Facilities: Case Study. JMIR *Med Inform.* 2018;6(2):e22. Published 2018 Apr 18. doi:10.2196/medinform.8403.
60. Naeem M, Alqasimi I. Unfolding and addressing the issues of electronic medical record implementation: evidence from public sector hospitals. *Inf Resour Manag J.* 2020;33(3):59-80. doi:10.4018/IRMJ.2020070103.
61. Noblin A, Cortelyou-Ward K, Cantiello J, et al. EHR implementation in a new clinic: a case study of clinician perceptions. *J Med Syst*. 2013;37(4):9955. doi:10.1007/s10916-013-9955-2.
62. Palvia P, Jacks T, Brown W. Critical Issues in EHR Implementation: Provider and Vendor Perspectives. *Communications of the Association for Information Systems.* 2015;36:36. doi:https://doi.org/10.17705/1CAIS.03636.
63. Pearce C, Bartlett J, Mcleod A, Eustace P, Amos R, Shearer M. Effectiveness of local support for the adoption of a national programme--a descriptive study. *Inform Prim Care.* 2014;21(4):171-178. doi:10.14236/jhi.v21i4.70.
64. Pine K, Mazmanian M. Artful and contorted coordinating: The ramifications of imposing formal logics of task jurisdiction on situated practice*. Acad Manage J.* 2016;60(1). doi:10.5465/amj.2014.0315.
65. Pohlmann S, Kunz A, Ose D, et al. Digitalizing Health Services by Implementing a Personal Electronic Health Record in Germany: Qualitative Analysis of Fundamental Prerequisites From the Perspective of Selected Experts. *J Med Internet Res.* 2020;22(1):e15102. Published 2020 Jan 29. doi:10.2196/15102.
66. Poss-Doering R, Kunz A, Pohlmann S, et al. Utilizing a Prototype Patient-Controlled Electronic Health Record in Germany: Qualitative Analysis of User-Reported Perceptions and Perspectives. *JMIR Form Res.* 2018;2(2):e10411. Published 2018 Aug 3. doi:10.2196/10411.
67. Rau E, Tischendorf T, Mitzscherlich B. Implementation of the electronic health record in the German healthcare system: an assessment of the current status and future development perspectives considering the potentials of health data utilisation by representatives of different stakeholder groups. *Front Health Serv*. 2024;4:1370759. Published 2024 May 10. doi:10.3389/frhs.2024.1370759.
68. Robertson A, Cresswell K, Takian A, et al. Implementation and adoption of nationwide electronic health records in secondary care in England: Qualitative analysis of interim results from a prospective national evaluation. *BMJ.* 2010;341(7778). doi:10.1136/bmj.c4564.
69. Scott JT, Rundall TG, Vogt TM, Hsu J. Kaiser Permanente's experience of implementing an electronic medical record: a qualitative study. *BMJ.* 2005;331(7528):1313-1316. doi:10.1136/bmj.38638.497477.68.
70. Ser G, Robertson A, Sheikh A. A qualitative exploration of workarounds related to the implementation of national electronic health records in early adopter mental health hospitals*. PLoS One*. 2014;9(1):e77669. Published 2014 Jan 16. doi:10.1371/journal.pone.0077669.
71. Sheehan OM, Greene RA, McKernan J, et al. Introduction of a Single Electronic Health Record for Maternity Units in Ireland: Outline of the Experiences of the Project Management Team. *JMIR Form Res*. 2023;7:e38938. Published 2023 May 12. doi:10.2196/38938.
72. Sheikh A, Cornford T, Barber N, et al. Implementation and adoption of nationwide electronic health records in secondary care in England: final qualitative results from prospective national evaluation in "early adopter" hospitals. *BMJ*. 2011;343:d6054. Published 2011 Oct 17. doi:10.1136/bmj.d6054.
73. Shield RR, Goldman RE, Anthony DA, Wang N, Doyle RJ, Borkan J. Gradual electronic health record implementation: new insights on physician and patient adaptation. *Ann Fam Med*. 2010;8(4):316-326. doi:10.1370/afm.1136.
74. Sidek YH, Martins JT. Perceived critical success factors of electronic health record system implementation in a dental clinic context: An organisational management perspective*. Int J Med Inform*. 2017;107:88-100. doi:10.1016/j.ijmedinf.2017.08.007.
75. Snowden A, Kolb H. Two years of unintended consequences: introducing an electronic health record system in a hospice in Scotland. *J Clin Nurs*. 2017;26(9-10):1414-1427. doi:10.1111/jocn.13576.
76. Stanczyk NE, Crutzen R, Sewuster N, Schotanus E, Mulders M, Cremers HP. Differences in Sociocognitive Beliefs between Involved and Noninvolved Employees during the Implementation of an Electronic Health Record System. *Perspect Health Inf Manag.* 2017;14(Spring):1c. Published 2017 Apr 1.
77. Standing C, Cripps H. Critical success factors in the implementation of electronic health records: A two-case comparison*. Syst Res Behav Sci.* 2013;32. doi:10.1002/sres.2209.
78. Strong DM, Volkoff O, Johnson SA, et al. A theory of organization-EHR affordance actualization. *J Assoc Inf Syst.* 2014;15(2)2. doi:10.17705/1jais.00353.
79. Takian A, Sheikh A, Barber N. We are bitter, but we are better off: case study of the implementation of an electronic health record system into a mental health hospital in England*. BMC Health Serv Res*. 2012;12:484. Published 2012 Dec 31. doi:10.1186/1472-6963-12-484.
80. Takian A, Sheikh A, Barber N. Organizational learning in the implementation and adoption of national electronic health records: case studies of two hospitals participating in the National Programme for Information Technology in England. *Health Informatics J.* 2014;20(3):199-212. doi:10.1177/1460458213493196.
81. Takian A. Envisioning electronic health record systems as change management: the experience of an English hospital joining the National Programme for Information Technology. *Stud Health Technol Inform*. 2012;180:901-905.
82. Threatt T, Pirtle CJ, Dzwonkowski J, Johnson KB. Using a custom mobile application for change management in an electronic health record implementation. *JAMIA Open*. 2019;3(1):37-43. Published 2019 Dec 16. doi:10.1093/jamiaopen/ooz048.
83. Tobler N, Colvin J, Rawlins NW. Longitudinal analysis and coping model of user adaptation. *J Comput Inf Syst*. 2016;57(2):97-105. doi:10.1080/08874417.2016.1183415.
84. Trocin C, Lee G, Bernardi R, Sarker S. How do unintended consequences emerge from EHR implementation? An affordance perspective. *Inf Syst J.* 2024. doi:10.1111/isj.12526.
85. Vadillo PC, Rojo ES, Garces A, Checton MG. Maximizing Healthcare Professionals' Use of New Computer Technologies in a Small, Urban Hospital's Critical Care Unit. J *Healthc Manag*. 2016;61(5):352-362.
86. van Offenbeek M, Vos J. An integrative framework for managing project issues across stakeholder groups*. Int J Project Manag.* 2015;34:10.1016/j.ijproman.2015.09.006. doi:10.1016/j.ijproman.2015.09.006.
87. van Offenbeek M, Vos J, Boonstra A. Understanding variation in subunit adoption of electronic health records: facilitating and constraining configurations of critical dependencies*. Eur J Inform Syst*. 2023;33:221-243. doi:10.1080/0960085X.2023.2225786.
88. Weston C, Wells-Beede E, Salazar A, et al. Patient-Centered Care Through Nurse Practitioner-Led Integrated Behavioral Health: A Case Study. *Public Health Rep.* 2023;138(1_suppl):36S-41S. doi:10.1177/00333549231152192.
89. Yung A. Adoption of Electronic Health Record System in Community-Based Physiotherapy Clinics: A Pilot Case Study. *Stud Health Technol Inform.* 2017;234:395-400.
90. Zandieh SO, Yoon-Flannery K, Kuperman GJ, Langsam DJ, Hyman D, Kaushal R. Challenges to EHR implementation in electronic- versus paper-based office practices. *J Gen Intern Med.* 2008;23(6):755-761. doi:10.1007/s11606-008-0573-5.
