# Supplementary material for: 25 Years of Electronic Health Record Implementation Processes: Scoping Review
Source: J Med Internet Res. 2025 Mar 3;27:e60077. doi: 10.2196/60077 (PMC11914847; doi:10.2196/60077)
Supplement: Multimedia Appendix 4 [file jmir_v27i1e60077_app4.docx]

**Multimedia Appendix 4.** Coding structure of selected data.

| **Theme** | **Code** | **Definition** | **Keywords** | **Example** |
| --- | --- | --- | --- | --- |
| Compliance | Government | References to National or Regional Government. | Government, Political | "However, private partners joined the project and the interdependencies between the inhouse ICT provider and the private partners were driven by MTMs, mainly in terms of contractual relationships with the regional government, which outsourced operations to all the main actors." [1] |
|  | Policy | Any reference to policy, government policy, organizational policy. | Policy, Political | "The case study organization had a strong centralized administrative and medical structure, which drove organizational goals and policies." [2] |
|  | Regulations | Mentions of regulations, often associated with policy | Regulations | "Political Regulations Many of the eHealth experts and HCPs were arguing in favor of a strong central solution for the problems." [3] |
|  | Vendors | Direct reference to vendors, and when vendor names are used e.g. EPIC. Mostly in line with early stages of implementation and system selection. | Vendors, System Selection | "Vendor Support both peer and technical support reportedly helped end-users to optimize their use of the EHRs and helped solve IT issues respectively." [4] |
| Collaboration | Communication | Communicating between team members and communicating EHR capabilities to users. | Communication, Dialogue, Awareness, Information campaigns | "VUMC leadership engagement and support throughout  the planning and implementation period ensured clarity of roles, strong communication practices and ultimately a successful outcome." [5] |
|  | Relationships | All mentions of relationships, also any mention of collaboration | Relationships, Collaboration | "A project leader emphasized that the relationship with the suppliers is ‘not only distant but even hostile, while buyer and the selected supplier are supposed to cooperate during the implementation phase’." [6] |
| Competence | Training | Any mention of training, largely staff training re system use | Training, Preparation | "These ﬁndings focused on the importance of training for both the success of the employee and medical group regarding EHR implementation." [7] |
|  | Support | User support, project support, any mention of support. | Support | "Over time, user support has become a higher priority than initial user training, and one that we propose adding to this category." [8] |
| Cost | Cost | Any mentions related to money. | Cost, Funding, Price, Pay | "The other category of goals were related to productivity and costs and included goals such as reducing turn-around time, increasing employee productivity, eliminating waste, and reducing redundancy." [9] |

## References

1. Cucciniello M, Guerrazzi C, Nasi G, Ongaro E. Coordination mechanisms for implementing complex innovations in the health care sector. *Public Manag Rev* 2015 May 13; 17(7):1040-1060.
2. Strong D, Volkoff O, Johnson S, Pelletier L, Tulu B, Bar-On I, Trudel J, Garber L. A theory of organization-EHR affordance actualization. *JAIS* 2014 Feb; 15(2):53-85.
3. Pohlmann S, Kunz A, Ose D, Winkler EC, Brandner A, Poss-Doering R, Szecsenyi J, Wensing M. Digitalizing health services by implementing a personal electronic health record in Germany: qualitative analysis of fundamental prerequisites from the perspective of selected experts. *J Med Internet Res* 2020 Jan 29; 22(1):e15102.
4. Mbwambo E, Mandari H. Acceptance of interoperable electronic health record (EHRs) systems: a Tanzanian e-health perspective. *J Int Technol Inf Manag* 2023 Jan 01; 32(1):96-121.
5. Kiepek W, Sengstack PP. An evaluation of system end-user support during implementation of an electronic health record using the model for improvement framework. *Appl Clin Inform* 2019 Oct 18; 10(5):964-971.
6. Boonstra A, van Offenbeek MA. Shaping a buyer's software selection process through tendering legislation. *Inf Syst* J 2017 Dec 14; 28(5):905-928.
7. Boswell RA Implementing electronic health records: implications for HR professionals. *Strateg HR Rev* 2013; 12(5):262-268.
8. deRiel E, Puttkammer N, Hyppolite N, Diallo J, Wagner S, Honoré J G, Balan JG, Celestin N, Vallès J S, Duval N, Thimothé G, Boncy J, Coq NRL, Barnhart S. Success factors for implementing and sustaining a mature electronic medical record in a low-resource setting: a case study of iSanté in Haiti. *Health Policy Plan* 2018 Mar 01; 33(2):237-246.
9. Deokar AV, Sarnikar S. Understanding process change management in electronic health record implementations. *Inf Syst e-Bus Manag* 2014 Jul 1; 14(4):733-766.
